# Active recovery affects the recovery of the corticospinal system but not of muscle contractile properties

**DOI:** 10.1371/journal.pone.0197339

**Published:** 2018-05-14

**Authors:** Louis-Solal Giboin, Ehsan Amiri, Raphael Bertschinger, Markus Gruber

**Affiliations:** 1 Sensorimotor Performance Lab, University Konstanz, Germany; 2 Faculty of Sport Science, Razi University, Kermanshah, Iran; University of Ottawa, CANADA

## Abstract

**Purpose:**

Active recovery is often used by athletes after strenuous exercise or competition but its underlying mechanisms are not well understood. We hypothesized that active recovery speeds-up recovery processes within the muscle and the central nervous system (CNS).

**Methods:**

We assessed muscular and CNS recovery by measuring the voluntary activation (VA) in the vastus lateralis muscle with transcranial magnetic stimulation (VA_TMS_) and peripheral nerve stimulation (VA_PNS_) during maximal voluntary contractions (MVC) of the knee extensors in 11 subjects. Measurements were performed before and after a fatiguing cycling time-trial, after an active and a passive recovery treatment and after another fatiguing task (1 min MVC). The measurements were performed a second time 24 h after the time-trial.

**Results:**

We observed a time × group interaction effect for VA_TMS_ (p = 0.013). Post-hoc corrected T-tests demonstrated an increased VA_TMS_ after active recovery when measured after the 1 min MVC performed 24 h after the time-trial (mean ± SD; 95.2 ± 4.1% vs. 89.2 ± 6.6%, p = 0.026). No significant effects were observed for all other variables.

**Conclusions:**

Active recovery increased aspects of central, rather than muscle recovery. However, no effect on MVC was seen, implying that even if active recovery speeds up CNS recovery, without affecting the recovery of muscle contractile properties, this doesn´t translate into increases in overall performance.

## Introduction

A faster recovery after competition or an exhaustive training session allows an individual to again perform sooner at his or her best. This is not only important in enabling a better overall performance in competitions that can last several days but also in limiting susceptibility to injury and overtraining [1–4]. However, the underlying mechanisms of performance recovery and recovery treatments are still not fully understood, which makes it difficult to tailor training programs for the purposes of speeding up recovery processes [4].

Active recovery is a popular recovery treatment used by athletes [5], and is defined as physical activity performed at low intensity following a fatiguing exercise. It has been proven to be effective in increasing subsequent performance compared to passive recovery [6–10]. It has been suggested that the higher performance after active, compared to passive recovery, is based on a faster recovery of muscle performance, as active recovery can affect muscle lactate metabolism and muscle oxygenation [7, 8, 11].

Beside the processes taking place within the muscle, it has been demonstrated that the central nervous system (CNS) plays an important role in the regulation of performance during exercise [12, 13]. The neuromuscular function following locomotor fatiguing exercise can remain impaired, from minutes up to days, depending on the duration of the task. The observed reduction in maximal voluntary force can be explained by a persisting presence of peripheral and central fatigue [14]. Interestingly, it appears that recovery over 48 h of impaired performance following intermittent sprint-exercise correlates better with the recovery of impaired neural drive to the muscles than with muscle damage markers [15]. Therefore, it could be suggested that the CNS plays an important role in the process of performance recovery [14–16]. Considering this, we hypothesized that active recovery may accelerate not only the recovery of the muscle itself, but also of the CNS, and thereby increase neuromuscular performance during the recovery period after an exhausting exercise.

Indeed, active recovery could possibly affect the recovery of CNS structures by several means. For example, during a fatiguing task, feedback from muscle afferents may reduce the neural drive and thus, reduce muscle activation [17]. Active recovery could speed up the clearance of the intramuscular metabolic perturbations induced by exercise [11], and thus limit the action of the afferent inhibitory feedback system on the neural drive.

Although the effect of active recovery has been studied mostly with short intermittent high intensity efforts so far [6–8], we selected a maximal continuous cycling exercise (time trial around 1 h) in the present study to induce a higher proportion of central fatigue [14, 18]. Following an active or passive recovery treatment, subjects performed a one minute long MVC. This maximal task was performed in order to observe the effect of the recovery treatment on performance. To observe the effect of active recovery on muscle and CNS recovery, we measured the potentiated twitch at rest (Ptw) and the voluntary activation (VA) of the vastus lateralis muscle. It has been shown that after a strenuous locomotor exercise, neuromuscular recovery can take several days to fully occur [14, 15] and that recovery of muscles and CNS may not follow the same trend over time [15]. Therefore, we reiterated our measurements 24 h after the end of the time-trial.

We hypothesized in the present study that active recovery, compared with passive recovery, improves parameters of muscle and CNS recovery as well as performance during a 1 min MVC test.

## Methods

### Participants

Experiments were approved by the ethics committee of the University of Konstanz and conducted in accordance with the declaration of Helsinki. 12 healthy male subjects were recruited and participated in the present study after signing the written informed consent. 1 subject suffered from a leg injury which was not related to the experiments and dropped out of the study. Therefore 11 subjects remained in the study (mean ± SD; age = 27.7 ± 4.3 years; height = 182.6 ± 3.9 cm; weight = 78.9 ± 5.9 kg). All subjects confirmed to be free of a lower limb injury at the time of the study and in the previous year and have no contra-indication for TMS. The subjects were asked to avoid caffeine and alcohol from the evening before until completion of testing.

### General experimental design

**Fig 1:**
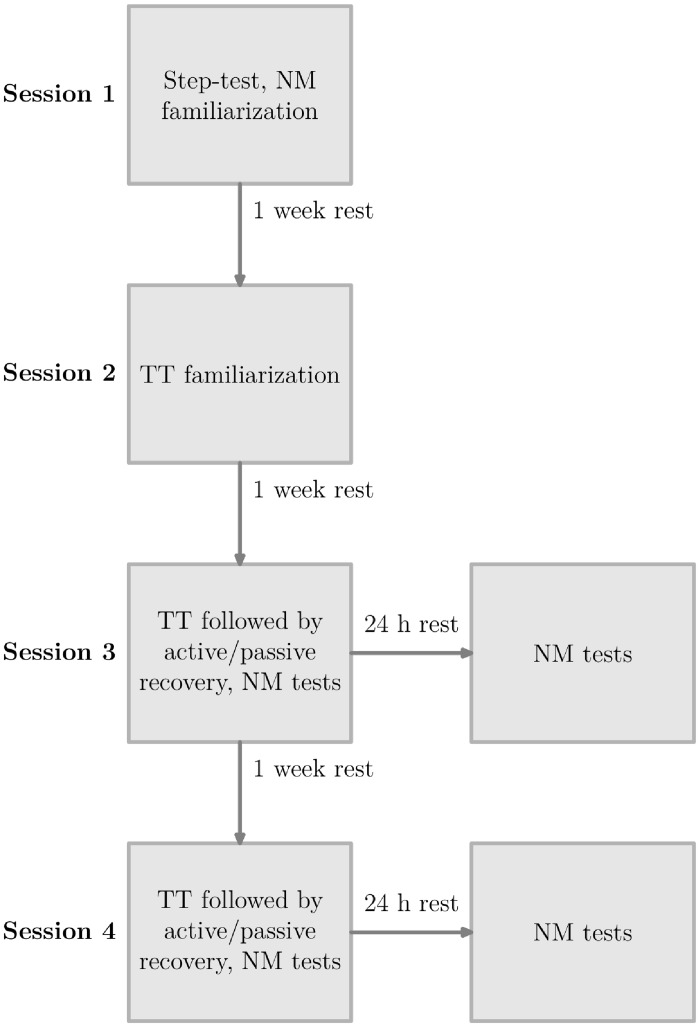
General experimental protocol. TT is the acronym for time-trial and NM the acronym for neuromuscular. The overall protocol consisted in 4 experimental sessions separated by 1 week of rest. In session 1, subjects performed a step-test and then were familiarized with NM tests. In session 2, subjects were familiarized with the time-trial. In session 3 and 4, the time-trial was followed by either the active or passive recovery treatment (random order). NM tests were performed before the time-trial, 2 min after the end of the time-trial, following the recovery treatment, after the 1 min MVC and again 24 h hours after the time-trial, before and after the 1 min MVC.

The subjects were asked to participate in 4 experimental sessions, separated by 1 week of rest. The experimental sessions for one subject were always performed the same day of the week and at the same time of the day (see Fig 1). During the first session, subjects performed a step test to assess the power output at their individual anaerobic threshold. After 20 min of rest, subjects were familiarised with the neuromuscular measurements and the MVC test. In the second session, subjects had to complete the time-trial with the goal of minimizing the overall ride time. For this familiarization time-trial, subjects were instructed to cycle with a constant power output, equivalent to or just below their anaerobic threshold power. A tactical debriefing took place following the end of the time-trial to advise subjects how to increase their time-trial performance for the next session. During the 3^rd^ and 4^th^ session, neuromuscular parameters were assessed at baseline prior to the time-trial. After the initial measurements, subjects then performed the time-trial. Upon time-trial completion, neuromuscular parameters were re-assessed (starting 2 min after the end of the time-trial). After post-time-trial measurements had been taken, subjects performed either an active or a passive recovery treatment in a randomised and counter-balanced order. Following recovery treatment, subjects went through the post-recovery neuromuscular measurements (starting 3 min after the end of the recovery treatment). Twenty-four hours after the 3rd and 4th session, subjects completed the post 24 h neuromuscular measurements.

### Cycling simulation

We used a custom-built cycling video simulation system, which consisted of a cycle ergometer driven by custom-coded cycling simulation software, that was faced toward a projection screen [19]. The cycle ergometer was a custom-made bicycle frame (made by the workshop of the university) coupled with a Cyclus2 brake system (RBM elektronik-automation GmbH, Leipzig, Germany). The projection on the screen consisted of a cyclist filmed during a ride on a specific road and any relevant information for the experiment (power output, map and gradient of the path, distance and time, current position of the cyclist on the map). The software controlled the speed of the video and the Cyclus2 braking system, forcing the subject to provide the power output necessary to cycle the virtual path. Moreover, the speed of the video was dependant on the subject’s current power output, giving the impression of cycling on the real path.

The software displayed an avatar on the road, just in front of the rider. This avatar was programmed to correspond with the performance of a previous time-trialof the same subject. The rider was able to see the current distance between himself and his avatar online throughout the time-trial. During the 3^rd^ experimental session, the avatar was paced with the speed that the subjects achieved during the familiarization session, in order to encourage them to beat their previous time. During the 4^th^ session, the avatar was paced with the speed that the subjects achieved during the 3^rd^ session. To assure similarity between the 3^rd^ and the 4^th^ session, subjects were instructed during the 4^th^ session to stay within −5/+5 m of the avatar from the 3^rd^ session. To circumvent possible effects from the following of an avatar on fatigue, the recovery treatment after the 3rd or 4th time-trial session counter-balanced among subjects.

### Step-test

The step-test was performed in power increments of 20 W every 3 min, starting at 100 W, until volitional exhaustion (mean ± SD; maximal power: 311 ± 62 W). We have used the official step-test protocol of the German Cycling Federation (BDR) documented in their youth program from 2012. Just before the end of each increment, blood was sampled at the ear lobe, in order to obtain a measure of blood lactate concentration (Lactate Pro 2, Arkray Factory inc.). Anaerobic threshold power was estimated as the power at which blood lactate concentration rose significantly (visual discrimination from the lactate blood concentration/power curve). We have used the method and the terminology proposed by Skinner & McLellan [20]. Maximal power reached during the step-test (maximal step-test power) corresponded to the maximal power reached by the subject at exhaustion. However, if the subject stopped at the beginning of a step, i.e. in the first min, the maximal step-test power corresponded to the power of the previous step. Pure water was supplied at the convenience of the subject throughout the whole experimental session.

### Time trial

**Fig 2:**
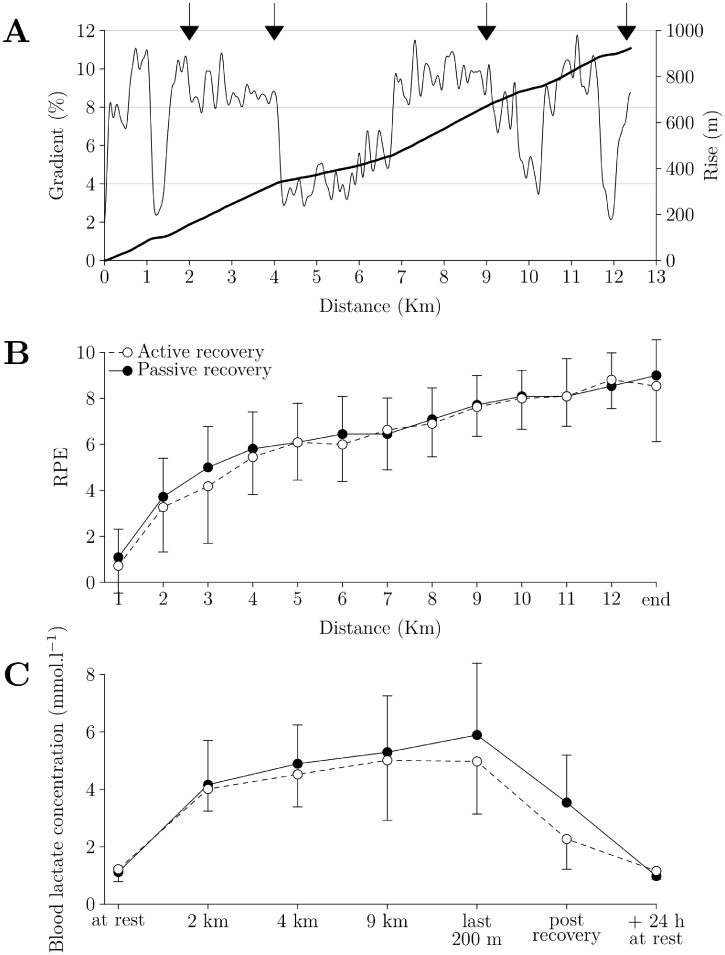
Gradient and rise of the time trial, blood lactate concentration and RPE values. A. The gradient (thin line, in %) and the rise (fat line, in m) of the time-trial are plotted against distance (in km). The black arrows correspond to blood lactate concentration measures. For B and C, the white dots and dashed line correspond to the time-trial followed by active recovery, the black dots and solid line to the time-trial followed by passive recovery. The error bars correspond to SD. For a better readability, significant differences over time are not indicated. B. RPE given at the end of each km and at the end of the time-trial. C. Blood lactate concentration (in mmol.l^-1^) measured at different time points.

A 9 min warm up that corresponded to the 3 first steps of the step-test protocol, preceded all time-trials. The time-trial was a simulation of the Alpine pass Flüela (western route, Switzerland). The time-trial distance was 12,397 m, with a rise of 923 m and an average gradient of 8.1% (see Fig 2A). To assess whether the time-trial induced a similar level of exertion during the 3rd and 4th experimental sessions, we measured blood lactate concentration at kilometre 2, 4, 9 and during the last 200 m of the session to estimate the level of exertion [21] (see Fig 2C). Heart rate was measured via an ANT+ heart rate belt (HRM1B, Xplova, Taipei City, Taiwan) and a Laptop with ANT+ Dongle. The Golden Cheetah Analysis Software (Version 3.3) was used to sample heart rate data in intervals of 1 Hz. Participants rated their perceived exertion (RPE; Borg 10 scale) every kilometre and at the end of the time-trial (see Fig 2B).

### Recovery treatment

Recovery treatments were performed immediately following the completion of the post time-trial neuromuscular measurements. The active recovery treatment consisted of cycling at 100 W at preferred pedalling cadence for a duration of 15 min. During passive recovery participants were asked to sit in a chair for the same time. Blood lactate concentration was measured at the end of both the active and the passive recovery treatment.

### Neuromuscular measurements

#### General procedure

Before the pre and post 24 h measurements, we measured blood lactate concentration at rest. Following the blood drawings, the right leg of each subject was prepared for the application of surface EMG sensors (Trigno wireless EMG system, Delsys Inc.) We placed the EMG electrodes according to SENIAM recommendations. The respective areas on the muscle bellies of the vastus lateralis (VL), rectus femoris, vastus medialis and biceps femoris (BF) were shaved, abraded with sand paper and cleaned with alcohol. EMG sensors were then applied to and taped to assure firm contact. EMG signals were amplified (× 1000), high-pass and low-pass filtered (20 ± 5 Hz and 450 ± 50 Hz respectively), sampled (4000 Hz) and registered on a computer through a Power 1401 interface (Cambridge Electronic Design) with the Signal software (Cambridge Electronic Design).

A mark was drawn with a waterproof pen on the right ankle, 2 cm higher than the line passing below the lateral malleolus, to ensure identical placement of the force transducer and knee angle throughout the entire experiment, as well as reapplication occurring 24 h later and the next week.

The subject was then seated in a custom-made chair that had been manufactured to measure isometric force during knee extensions. Participants were strapped at the chest and hip level, with the right ankle tightly fixed to a force transducer (Model 9321A, Kistler, Wintherthur, Switzerland) holding the knee at around 90 ° of flexion in a non-compliant way. Force signals were sampled and registered with the same interface and software as EMG signals.

To stimulate the femoral nerve (Stimulator DSH7, Digitimer), a cathode (copper, circular, 2 cm diameter, wrapped in a water soaked sponge) was taped in the femoral triangle and an anode (copper, 7 × 5 cm, wrapped in a soaked sponge) was taped over the gluteus maximus. Positions of both stimulation electrodes were marked on the skin with a waterproof pen to ensure similar and fast electrode repositioning after the time-trial and the recovery treatments. To ensure that the cathode was positioned as close as possible to the femoral nerve throughout the entirety of the experiment, an investigator held a small sandbag over its position and applied pressure. The pulse duration of the electrical stimulation was set to 1 ms and the intensity of the stimulation was gradually increased until reaching Mmax in the VL at rest and the maximum un-potentiated twitch at rest of the knee extensors, without any visual detectable motor response in the BF EMG. The intensity required for reaching Mmax was multiplied by 1.5 and remained constant during the entire experiment (final intensity: 73 ± 13 mA).

The subject completed a warm up of around 15 contractions, with gradual increases in intensity and rest periods, until reaching an intensity equal to around 90% of the perceived maximal voluntary contraction (MVC). After a rest of 2 min, the subject performed an initial MVC trial, followed by a second trial occurring another 2 min later. In the second trial the force did not increase more than 5% in none of the subjects, therefore a third trial was not performed. All MVCs were completed with a force plateau of 2–3 s. The investigator encouraged and motivated the subject verbally before and during all maximal MVC trials.

After the initial MVCs, we searched for the best position of the transcranial magnetic stimulation (TMS) coil to induce a motor evoked potential (MEP) in the VL during a 10% MVC contraction. The coil (MC-B70, MagVenture) and the stimulator (MagPro R30, MagVenture) was set to produce biphasic single pulse (butterfly-coil oriented in the posterior anterior axis of the head). The optimal position of the coil was drawn directly on the scalp to ensure identical coil positioning throughout the experiment. The stimulation intensity was set to evoke the largest possible MEP (~10–50% Mmax amplitude) in the VL, with the lowest response in the BF (visual control of BF EMG) at 10% MVC (90–100% maximal stimulation output).

**Fig 3:**
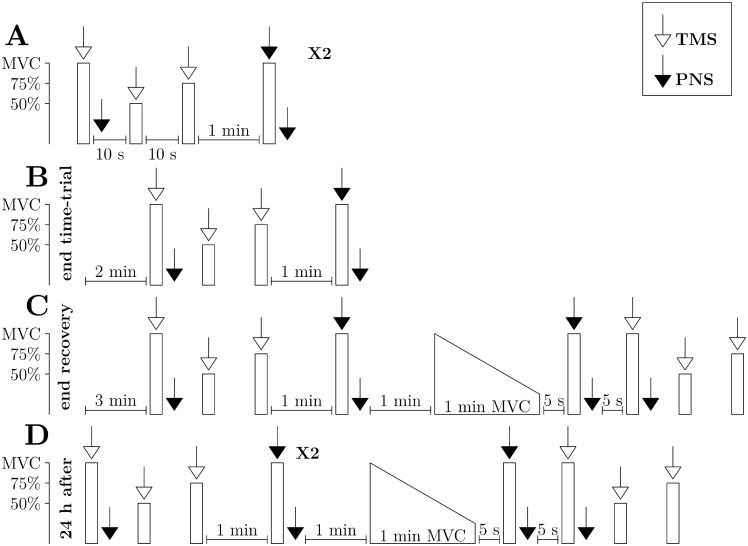
Protocols of neuromuscular measurements. White arrows indicate a TMS measurement, black arrows indicate a PNS measurement, white column indicate voluntary contractions up to 50, 75 or 100% MVC, and white trapeziums indicate a 1 min long MVC. A. Pre measurements, × 2 means that the set of measurements (VA_TMS_ and VA_PNS_) was repeated 2 times. B. Post measurements. C. Post recovery measurements. D. Post 24 h measurements, × 2 means that the set of measurements (VA_TMS_ and VA_PNS_) was repeated 2 times.

**Fig 4:**
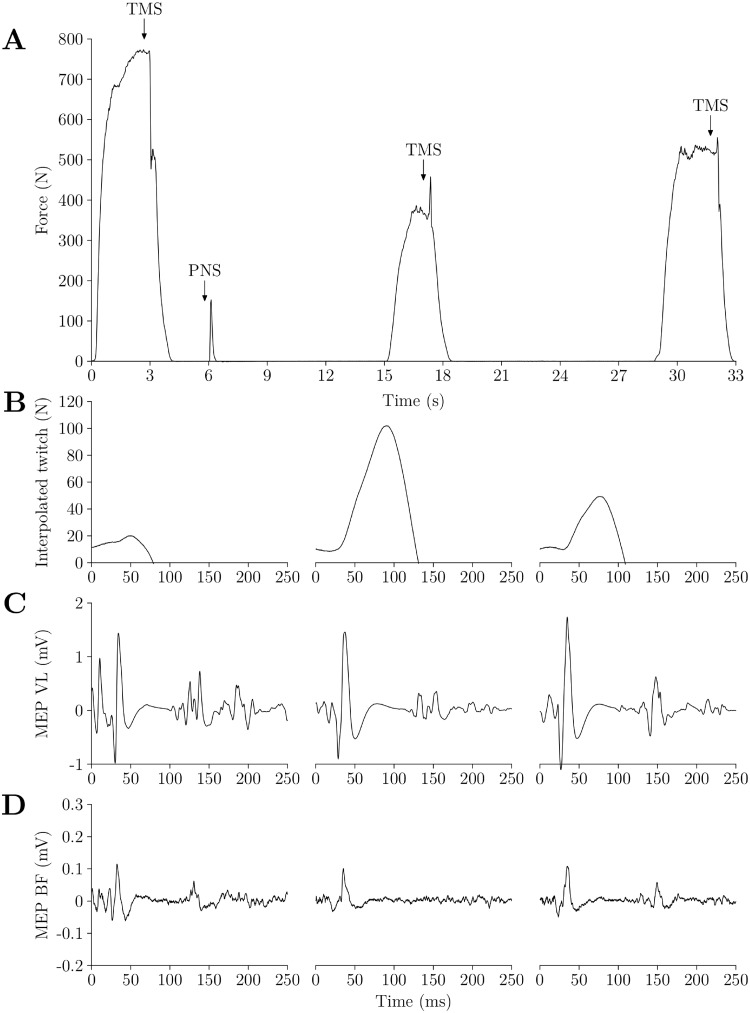
Example of VA_TMS_ procedure in one subject. A. Force curve (in N) during the VA_TMS_ procedure. Subject is asked to perform a 100% MVC, a 50% MVC and a 75% MVC. Contractions are separated by around 10 s. TMS is performed at the plateau of each contraction. PNS is performed after the 100% MVC to measure Ptw. B, C, D. Force trace of the interpolated muscle twitch (in N), EMG trace of the MEP in the VL and BF muscle (in mV) during the 100%, 50% and 75% MVC. Time point 0 ms corresponds to TMS.

During the experiment, we measured voluntary activation via 2 different methods, TMS (VA_TMS_) and peripheral nerve stimulation (VA_PNS_). VA_TMS_ and VA_PNS_ were always measured during separated muscle contractions (see Fig 3). We measured VA_PNS_ following the interpolated twitch method protocol [22], i.e. by stimulating the femoral nerve during the force plateau of MVC and at rest 2 s after the end of the contraction and VA_TMS_ following the guidelines of Sidhu and colleagues [23] for the quadriceps, i.e. TMS at the plateau of MVC, of a 50% MVC and 75% MVC contraction (see Fig 4), with 3 muscle contractions being separated by 10 s of rest. During the VA_TMS_ protocol, 2 s after the first MVC, we stimulated the femoral nerve with peripheral nerve stimulation to obtain a potentiated twitch at rest (Ptw) in order to estimate peripheral fatigue as soon as possible in the post cycling and post recovery neuromuscular measurements since VA_TMS_ was measured before VA_PNS_ (Fig 3). Contrary to Sidhu and colleagues [23] we interspaced VA_TMS_ and VA_PNS_ measurements by one minute in order to ensure that the MVC required for the VA_PNS_ procedure was not affected by potential fatigue induced by the earlier VA_TMS_ procedure. During all procedures involving a muscular contraction, we provided visual feedback of all the target forces on a computer screen that was placed in front of the subjects.

#### Neuromuscular measurements

Pre time-trial measurements: we measured VA_TMS_ first. Then, 1 min after the last contraction of the VA_TMS_ procedure, we measured VA_PNS_. After 2 min of rest, this sequence was repeated (Fig 3A). Post time-trial measurements: 2 min after the end of the time-trial, we measured VA_TMS_, and 1 min later we measured VA_PNS_ (Fig 3B). Post recovery treatment measurements: we measured VA_TMS_, and 1 min later we measured VA_PNS_. One minute of rest later, the subject had to perform a 1 min MVC, with the following instructions: "Go full gas, reach MVC and maintain it for 1 min". Five seconds after the end of the 1 min MVC, we measured VA_PNS_ and 5 s later, VA_TMS_ (Fig 3C). This sequence of measurements after the 1 min MVC seems to be reasonable, as central fatigue may recover faster after a 1 min MVC than after a 1 h time-trial. Post 24 h measurements: we measured VA_TMS_ and VA_PNS_ twice, as in the pre-measurements. One min later, the subject had to perform a 1 min MVC and we measured 5 s later VA_PNS_ and 5 s later VA_TMS_ (Fig 3D).

### Analysis

MVC was determined as the maximal value of the force measured during the maximal isometric leg extensions. Ptw values corresponded to the maximal amplitude of the potentiated twitch at rest.

For the pre and the beginning of the post 24 h neuromuscular measurements, MVC, VA_TMS_, VA_PNS_ and Ptw corresponded to the highest values measured during both trials. For the post time-trial and post recovery measurements, MVC and Ptw were determined only during the first MVC trial, which we obtained during the VA_TMS_ protocol. After the 1 min MVC (in the post recovery and post 24 h neuromuscular measurements), we averaged the MVCs and Ptws obtained during VA_PNS_ and VA_TMS_ procedures. It must be noted that one subject could not perform the VA_TMS_ measurement during one session, due to stomach issues just after the end of the time-trial. However, he was able to perform the following VA_PNS_ procedure. Therefore, when considering the effect of training over time, the VA_TMS_, MVC and Ptw measures were analysed only for the remaining 10 subjects.

To quantify the effort produced during the 1 min MVC, we measured the mean force and the peak force (i.e. maximum amplitude) produced during the 1 min MVC.

We measured the MEP amplitude in the VL (normalized to Mmax) and in the BF (non-normalized) during the VA_TMS_ procedure. We also indicated the value of the estimated resting twitch, which is calculated as the y-intercept of the linear relation between the voluntary activation force amplitude and the interpolated twitch amplitude [24]. Additionally, we measured the correlation coefficient (Pearson's r) of this linear regression to give the reader an estimate of the success of the VA_TMS_ procedure [25].

Mean power and mean standard deviation corresponded to the mean of the power output and the standard deviation of the power respectively during the time-trial followed by the active or the passive recovery treatment. Mean heart rate corresponded to the averaged heart rate measured during the entire time-trial.

### Statistics

Shapiro-Wilk normality tests were performed on each data set. Two-way, within-subject ANOVAs were completed on lactate, RPE, MVC, Ptw, VA_PNS_, VA_TMS_ values. Mauchly's tests of sphericity were performed, and when significant, a Greehouse-Geisser correction was used. When relevant, post hoc tests were completed using a pairwise comparison with paired T-Tests and a Holm-Bonferroni correction. Post-hoc tests for time effects were conducted against each time point, with data from both treatments pooled. To better understand group × time effect, *t*-tests were conducted between groups at each time point.

Paired T-tests were used to compare mean power, mean standard deviation, time, mean heart-rate during the time-trial, mean and peak force during the 1 min MVC, with the active or passive recovery treatment. We calculated Cohen’s d for paired t-test (d = mean of paired difference /sd of paired difference).

## Results

### Neuromuscular measurements

**Fig 5:**
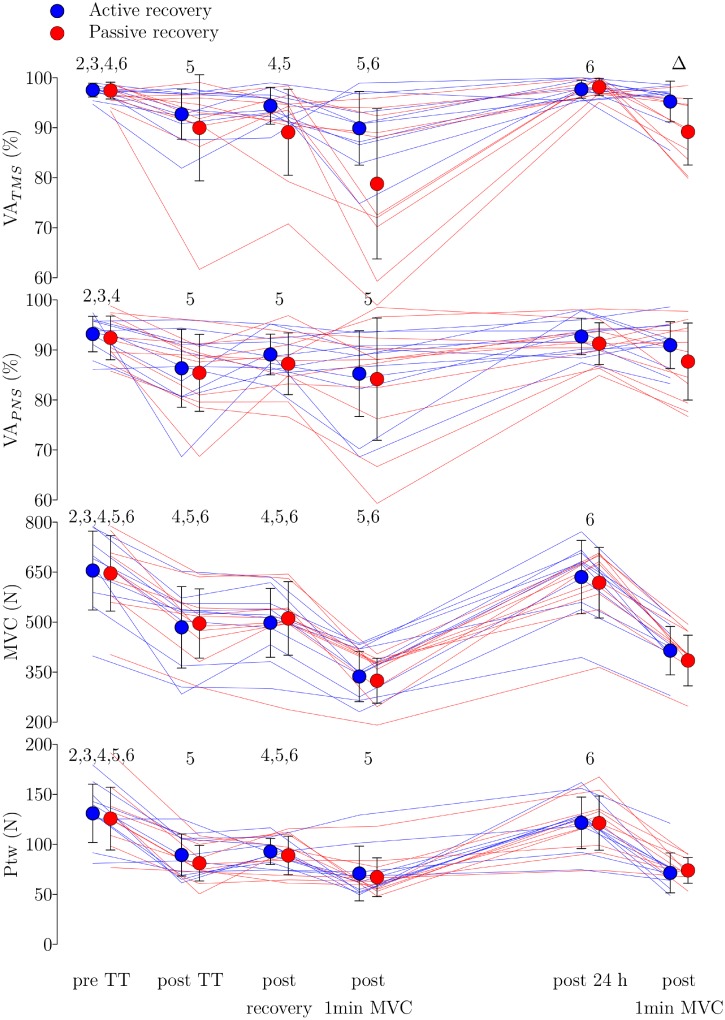
Neuromuscular results. Measures of VA_TMS_ (in %), VA_PNS_ (in %), MVC (in N) and Ptw (in N) across time. The error bars correspond to SD. The blue dots correspond to the mean measures done in the experimental session with active recovery and the red dots to the session done with passive recovery. Thin blue and red lines correspond to individual data obtained in the session with active recovery and passive recovery respectively. The Delta symbol represents a post-hoc difference between group with a corrected p-value = 0.026. The numbers above each pooled time point correspond to the result of post-hoc tests for time. The display of a number corresponds to a significant difference (p < 0.05) with the associated time point (1 corresponds to pre time-trial, 2 to post time-trial, 3 to post recovery, 4 to post 1 min MVC, 5 to post 24 h, and 6 to the second post 1 min MVC).

As displayed in Fig 5, there was a time effect but no group or group × time effect measured for VA_PNS_ (p < 0.001, p = 0.35 and p = 0.94 respectively), MVC (p < 0.001, p = 0.55 and p = 0.46) and Ptw (p < 0.001, p = 0.25 and p = 0.19). We found no difference in the mean force exerted by the participants during both 1 min MVCs, nor when performed the same day than the time-trial (active recovery = 297 ± 103 N; passive recovery = 300 ± 84 N, p-value = 0.87, Cohen's d = -0.051) nor 24 h after the time-trial (active recovery: 388 ± 114 N; passive recovery: 373 ± 100 N, p-value = 0.21, Cohen's d = 0.4).

However, for VA_TMS_, we observed a time (p < 0.001), group (p = 0.022) and group × time effect (p = 0.013). Post hoc tests with Holm-Bonferroni correction revealed that the interaction effect could partly be explained by a higher VA_TMS_ with the active recovery treatment measured after the 1 min MVC performed 24 h after the time-trial (95.2 ± 4.1% vs. 89.2 ± 6.6%, t_9_ = 3.8, p = 0.026, Cohen's d = 1.2, see Fig 5). The neuromuscular values measured 24 h after the time-trial (post 24 h) were not significantly different from the pre values measured before the time-trial, with the exception of the Ptw, which was significantly lower (Fig 5).

### Control variables for the VA_TMS_ measurements

The estimated resting twitch (in N), the estimated resting twitch correlation coefficient, the MEP obtained in the VL at 100, 75 and 50% MVC (normalized to Mmax), and the MEP in the BF at 100, 75 and 50% MVC (in mV) are displayed in Table 1. It must be noted that a treatment effect was observed for the estimated resting twitch (p = 0.042), explained by slightly lower values for the passive recovery treatment (post-hoc T-tests were not significant).

**Table 1:** VA_TMS_ control variables.

Time	Treatment	pre TT	post TT	post recovery	post 1 min MVC	post 24 h	post 1 min MVC	2 ways ANOVA		
								time	treatment	interaction
ERT	active	85 (39)	47 (15)	49 (16)	51 (12)	73 (31)	60 (21)	0.005	0.042	0.31
	passive	78 (46)	34 (14)	37 (16)	40 (19)	75 (30)	53 (22)			
ERT corr	active	-0.96 (0.03)	-0.95 (0.03)	-0.95 (0.06)	-0.93 (0.09)	-0.94 (0.05)	-0.92 (0.09)	0.19	0.65	0.29
	passive	-0.96 (0.02)	-0.94 (0.07)	-0.87 (0.17)	-0.89 (0.14)	-0.96 (0.03)	-0.98 (0.03)			
MEP VL 100% MVC	active	33 (13)	25 (11)	30 (17)	28 (12)	31 (18)	33 (18)	0.38	0.1	0.26
	passive	31 (18)	34 (18)	34 (12)	43 (15)	29 (10)	36 (15)			
MEP VL 75% MVC	active	36 (15)	36 (18)	35 (15)	37 (15)	32 (15)	36 (21)	0.78	0.56	0.86
	passive	35 (25)	41 (23)	40 (12)	38 (24)	36 (17)	37 (20)			
MEP VL 50% MVC	active	36 (13)	36 (15)	33 (16)	27 (15)	35 (16)	30 (21)	0.22	0.48	0.64
	passive	35 (17)	40 (21)	42 (18)	33 (20)	38 (17)	34 (23)			
MEP BF 100% MVC	active	0.47 (0.6)	0.24 (0.27)	0.29 (0.32)	0.17 (0.22)	0.35 (0.35)	0.26 (0.29)	0.02	0.33	0.35
	passive	0.64 (0.59)	0.45 (0.49)	0.42 (0.27)	0.22 (0.14)	0.49 (0.45)	0.19 (0.18)			
MEP BF 75% MVC	active	0.24 (0.25)	0.11 (0.04)	0.12 (0.06)	0.12 (0.09)	0.15 (0.1)	0.13 (0.08)	0.05	0.08	0.37
	passive	0.3 (0.25)	0.25 (0.16)	0.18 (0.09)	0.13 (0.08)	0.21 (0.19)	0.17 (0.14)			
MEP BF 50% MVC	active	0.13 (0.07)	0.1 (0.07)	0.12 (0.1)	0.12 (0.1)	0.11 (0.05)	0.1 (0.06)	0.51	0.27	0.77
	passive	0.17 (0.1)	0.16 (0.1)	0.13 (0.09)	0.15 (0.11)	0.12 (0.08)	0.14 (0.13)			
MEP BF in % VL 100% MVC	active	31 (61)	22 (25)	27 (41)	19 (35)	26 (30)	20 (29)	0.016	0.81	0.52
	passive	42 (40)	34 (41)	29 (18)	16 (19)	33 (31)	10 (10)			

The estimated resting twitch (ERT; in N), the Pearson's r coefficient of the variables used to calculate the estimated resting twitch (ERT r), the amplitude of MEP in the VL (in % Mmax) during the 100%, 75% and 50% MVC, the amplitude of MEP in the BF (in mV) during the 100%, 75% and 50% MVC, and the amplitude of MEP BF in % VL 100% MVC (i.e. the amplitude of MEP in the BF expressed in % MEP in the VL during the 100% MVC) are displayed in function of the recovery treatment and the time of the measurement. TT is the acronym for time-trial.

### Reproducibility of the time-trial

There was no statistical difference between the time-trial followed by the active or passive recovery treatment for the duration of the time-trial (63 min 1 s ± 14 min 45 s and 62 min 52 s ± 14 min 59 s respectively, p = 0.29), mean power (258 ± 63 W and 260 ± 65 W, p = 0.22), mean power standard deviation (26 ± 9 W and 23 ± 4 W, p = 0.23), pedalling cadence (75 ± 11 rpm and 75 ± 11 rpm, p = 0.53) and mean heart rate (165 ± 9 bpm and 167 ± 9 bpm, p = 0.48).

For the RPE and blood lactate concentration (see Fig 2B and 2C respectively), there was a time effect (p < 0.001 for both), but no group effect (p = 0.39 and 0.25 respectively) or time × group interaction (p = 0.54 and 0.10).

## Discussion

In the present study, we observed that active recovery, compared to passive recovery, prevented the reduction of VA_TMS_ after the 1 min MVC performed 24 h after the time-trial. However, active recovery had no more effect than passive recovery on the recovery of Ptw and on the performance during MVCs.

### Active recovery affected central but not peripheral recovery

Active recovery, when compared to passive recovery, had a large effect on VA_TMS_ but no effect on VA_PNS_ and Ptw. The relevance of active recovery on VA_TMS_ is strengthened by the fact that its effect size is comparable to the one observed for the decreased VA_TMS_ after the time-trial compared to VA_TMS_ before the time-trial in the active recovery session (VA_TMS_ of 97.5 ± 1.4% pre time-trial vs. 92.7 ± 5% post time-trial, d = 1.13). We suggest that, in our experimental context, active recovery increased the recovery of corticospinal voluntary activation but not the recovery of the whole CNS voluntary activation or of muscle contractile properties. This result is strengthened by our control measurements: i) The time-trial induced the same amount of fatigue in the 2 experimental sessions, and therefore cannot explain the observed differences in VA_TMS_. ii) The control variables of the VA_TMS_ measurements showed no variation across time or between treatment that was similar to the one observed for VA_TMS_ and therefore cannot explain the observed difference of VA_TMS_ [25]. Thus, we conclude that the interaction effect observed for VA_TMS_ was most probably the result of a difference in corticospinal voluntary activation of muscles indicating that active recovery possibly accelerated the recovery of corticospinal structures when compared to passive recovery.

### Active recovery did not increase performance

Despite the effect of active recovery on VA_TMS_, we could not observe an effect on maximal performance as measured during MVCs and the 1 min MVC. This observation appears to be contradictory with the abundant evidences that the voluntary activation level is an important contributor to force production following exercise [14]. How to explain the lack of effect on maximal performance? It has to be mentioned that, in the present study, we detected a difference of VA_TMS_ only during measurements while performance was heavily impaired (i.e. after the 1 min MVC), thus in situations where we also observed a large amount of peripheral fatigue. We propose that, due to the orderly motor units recruitment [26], the difference of VA_TMS_ induced by the recovery treatments mostly affected the recruitment and/or firing rate of the high threshold motoneurons of the pool. During the 1 min MVC, these motor units were presumably fatigued in the first few seconds of the task [27], and after that, were not contributing anymore to force production. Then, their activation with active recovery, or their lack of activation with passive recovery, would not change the force output. Such effect can explain why a larger corticospinal voluntary activation could not induce a larger mean force in the 1 min MVC or maximal force in the following MVC. In a previous study, after a fatiguing task, we also observed an attenuated reduction in VA_TMS_, probably induced by brain stimulation, but not an attenuated reduction in Ptw. Similarly to the present study, the attenuation in VA_TMS_ reduction was not accompanied by an immediate effect on MVC [28]. Thus, in the present experimental context, the main limiting factors of performance following the time-trial seem to have more peripheral than central origin.

In contrast to the results of the present study, it has been discussed that the CNS could be an important player in overall performance recovery [14–16]. However, the studies mentioned present mainly indirect and sometimes contradictory results. For example, in several studies, the time course recovery of MVC follows more the recovery of neural than muscles markers, indicating that performance might be driven more by central than peripheral substrates [14, 15]. On the other hand, Thomas and colleagues have shown that after a simulated football game, VA_PNS_ and VA_TMS_ recovered faster than Ptw and MVC, indicating that muscle function recovery primarily depended on peripheral aspects of fatigue [29]. The mechanisms regulating performance recovery seems to be highly specific to the previous fatiguing task [14]. In the present study, we were able to target specifically the corticospinal performance recovery. This particular situation offered us the possibility to assess with direct observation the role of the CNS in maximal force production following a fatiguing task. The lack of effect on performance from the larger corticospinal voluntary activation suggests that after a cycling time-trial or a 1 min MVC, the main limitation in overall performance recovery is more muscular than central.

Since we only made measurement during maximal efforts, we cannot conclude about the possible effects of a larger VA_TMS_ on submaximal tasks. We propose that a submaximal task could benefit more from a larger VA_TMS_, since in this situation, the relative main contributor to muscle fatigue may be more central than peripheral [14].

### Limitations

We found an effect of active recovery on VA_TMS_ but not on VA_PNS_. We have concluded that active recovery affected more corticospinal structures than the whole CNS. However, such conclusions must be considered cautiously as they are not free from methodological pitfalls. Indeed, the difference of effect between the two VA procedures may come from the possible sigmoidal relationship between the excitation of the motoneuron pool and the size of the interpolated twitch during VA_PNS_ [30] and the more linear relationship observed during VA_TMS_ [23, 24, 31, 32]. Additionally, the recovery treatment may have had a different effect on the nervous structures and synergist or antagonist muscles that are activated in different proportions during VA_TMS_ and VA_PNS_ [31]. Moreover, the different ways to produce interpolated twitches during VA_PNS_ and VA_TMS_ may also have been affected differently by recovery treatments, e.g. spinal reflexes that may have influenced the twitch size during VA_PNS_ [30].

We have used an active recovery treatment with a fixed power (i.e. 100 W). A treatment tailored to the aerobic capacity of each subject could have provided less variation in the effect of active recovery on neuromuscular performance. However, the 100 W treatment was well under the anaerobic threshold for all the subjects, and therefore we do not expect a negative influence on the recovery processes following the treatment.

In general, due to the complexity of the present study with many variables measured over several time points and a rather low number of subjects, statistics can be biased. On the one hand statistical power could have been too low to detect existing differences, and on the other hand the high number of comparisons could have provided false positive results. Therefore, the effect of active recovery on corticospinal voluntary activation should be confirmed in a further study tailored to the specific needs of this research question.

### Conclusion

The present study shows that an active recovery treatment performed after an exhausting endurance cycling task can have an effect on corticospinal structures without having an effect on muscle contractile properties or performance. This indicates that in our experimental paradigm (i.e. after a long duration cycling uphill time-trial), the main limiting factor of overall performance recovery takes place more at peripheral than central level. The results of the present study support the need of a better understanding of the underlying mechanisms of recovery treatments in order to tailor the recovery strategy specifically to the previous and upcoming effort.

Given that an active recovery treatment has the capacity to accelerate the recovery or to prevent the reduction of corticospinal voluntary activation after a fatiguing task, its application would be optimal in a situation where performance is mainly limited by central components. In that context, Husmann et al. have shown that knee extensor MVC following a short duration rowing time-trial in elite rowers was mostly limited by an impaired central drive [33]. Therefore, we propose that active recovery could have a greater effect in a short duration rather than in a long duration exhausting exercise. We suggest that a rowing time-trial, as used by Husmann et al. or a similar exercise could provide an excellent experimental paradigm to further study the role of the CNS in overall performance recovery.

## Abbreviations

CNS: central nervous system
MVC: maximal voluntary contraction
Ptw: potentiated muscle twitch at rest
TMS: transcranial magnetic stimulation
VA: voluntary activation
VA_PNS_: VA measured with peripheral nerve stimulation
VA_TMS_: VA measured with TMS
